# Effect of Calafate (*Berberis microphylla*) Intake on Plasma Atherogenic Indices in Rats With High‐Sucrose Diet–Induced Obesity

**DOI:** 10.1155/jnme/8845471

**Published:** 2026-01-02

**Authors:** Carla Guzmán-Pincheira, Gabriel Araujo-Silva, Raul Sánchez-Gutiérrez

**Affiliations:** ^1^ Escuela Nutrición y Dietética, Facultad de Ciencias de la Rehabilitación y Calidad de Vida, Universidad San Sebastián, Lientur 1457, Concepción, Chile, uss.cl; ^2^ Organic Chemistry and Biochemistry Laboratory, State University of Amapá (UEAP), Macapá, 68900070, Amapá, Brazil, ueap.edu.br; ^3^ Center of Excellence in Translational Medicine-Scientific and Technological Bioresource (CEMT-BIOREN), Universidad de La Frontera, Temuco, Chile, ufro.cl

**Keywords:** atherogenic index, calafate berry, HDL cholesterol, high-sucrose diet, polyphenols

## Abstract

Cardiovascular diseases (CVDs) are multifactorial conditions strongly linked to elevated obesity rates and sedentary lifestyle; among the contributing mechanisms, alterations in lipid metabolism—particularly dyslipidemia—play a central role in their pathogenesis. The present study aimed to evaluate the effects of calafate (*Berberis microphylla*) supplementation on the lipid profile and atherogenic indices in rats with obesity induced by a high‐sucrose diet. Sprague Dawley rats received a high‐sucrose diet and were supplemented with 350 mg/kg/day of freeze‐dried calafate for 10 weeks (BM group). Dietary intake, plasma glucose, lipid parameters, and cardiovascular risk indices were assessed. Compared to controls, calafate‐supplemented rats showed a significant increase in HDL‐c and total cholesterol, with the latter showing a 166% elevation. Additionally, calafate intake was associated with a marked decrease in the atherogenic index and the glucose–triglyceride index, suggesting an improvement in cardiovascular risk markers. These findings support the potential cardioprotective and antithrombotic properties of calafate, reinforcing its relevance as a functional food.

## 1. Introduction

Cardiovascular diseases (CVDs) are currently considered a global epidemic, with an estimated prevalence of 423 million cases [1] and approximately 20.5 million deaths per year [2]. Although multifactorial in origin, CVDs are strongly associated with high rates of obesity and sedentary behavior. Among the contributing factors, alterations or disturbances in lipid metabolism, particularly those leading to dyslipidemia, play a main role in the early pathophysiological processes underlying these conditions [3]. Consequently, the studies and developments of new effective therapies to modulate lipid profiles and reduce cardiovascular risk (CVR) remain a major area of scientific interest.

Dietary models based on high‐sucrose intake have been widely used to induce obesity and related metabolic alterations in rodents, particularly in rats [4]. This approach mimics essential features of human obesity by promoting increased energy consumption, body weight gain, and disruptions in lipid metabolism. High‐sucrose diets have been shown to elevate plasma triglycerides (TG) and total cholesterol, and reduce high‐density lipoprotein cholesterol (HDL‐c) levels, thereby contributing to the development of an atherogenic profile [4, 5]. Moreover, these changes are often accompanied by elevated atherogenic indices, such as Castelli’s Risk Indices and the TG–glucose (TyG) index, which are reliable markers of CVR. Although the impact on body mass can vary depending on strain and diet composition, rats subjected to high‐sucrose diets typically show increased caloric intake and adiposity. Given their reproducibility and physiological responsiveness, rats represent a suitable and validated model for evaluating dietary interventions targeting lipid homeostasis and CVR [6].

The distribution of cholesterol among plasma lipoprotein fractions is used as a classical marker of atherosclerosis risk [7–9]. HDL‐c is known to exert a protective mechanism for atherosclerotic lesions, whereas low‐density lipoprotein cholesterol (LDL‐c) is considered a primary atherogenic factor [9]. Despite therapeutic strategies aimed at lowering LDL‐c, specifically related to lipid‐lowering pharmacological treatments such as statins, evidence indicates that CVR is not greater than 50% of patients, so their clinical efficacy is relative and does not eliminate most of the residual risk [10]. Meta‐analyses by the Cholesterol Treatment Trialists’ (CTT) Collaboration have found that for every 39 mg/dL reduction in LDL‐c, the relative risk of major cardiovascular events decreases by 20%–22%, indicating that even with intensive treatment, there remains a considerable risk for the patient [11]. This scenario has prompted the search for complementary therapies, among which dietary bioactive compounds such as polyphenols stand out. Their antioxidant, anti‐inflammatory, and endothelial function–modulating effects could offer additional benefits beyond traditional lipid reduction [12, 13].

Calafate (*Berberis microphylla*), a native berry from the Patagonian region of Chile and Argentina, is considered a rare species and a promising source of bioactive secondary metabolites, such as flavonoids and anthocyanins [14–16]. These activity compounds are also found in *Berberis* leaves and are associated with antioxidant and anti‐inflammatory properties [17]. In a murine model, Olivares‐Caro et al. reported that calafate extract inhibited TNF‐α gene expression in macrophages and reduced lipid peroxidation, also modulating markers such as thrombomodulin and adiponectin after chronic administration in animals fed a high‐fat diet [18, 19]. These effects correlate with the polyphenol profile, including anthocyanins, hydroxycinnamic acids, proanthocyanidins, and flavonols [20].

Despite growing interest in the bioactive properties of calafate [14, 15, 17, 18], few studies have evaluated its effects on atherogenic indices, especially under conditions of prolonged high‐sucrose intake. Previous research by our group demonstrated its impact on the lipid profile [16] but did not explore composite CVR markers. Moreover, most studies have employed high‐fat diets, leaving a gap in understanding their effects in models mimicking excessive sugar consumption. These limitations highlight the need for experimental approaches that assess both conventional lipids and sensitive risk indices to clarify the biological properties of calafate.

In light of these considerations, the present study aimed to evaluate the effect of calafate (*B. microphylla)* intake on plasma lipid profile and atherogenic indices in rats with obesity induced by a high‐sucrose diet. We hypothesized that calafate supplementation could improve lipid parameters and reduce composite CVR markers, such as Castelli’s Risk Indices and the TyG index, under conditions of diet‐induced metabolic stress.

## 2. Materials and Methods

### 2.1. Animals

Male Sprague Dawley rats (6–7 weeks old), obtained from the Regional Center for Advanced Studies in Life Sciences at the University of Concepción, Chile, were used in this study. Animals were maintained under controlled laboratory conditions: 12‐h light/dark cycle, ambient temperature of 25°C ± 1°C, and 60% relative humidity. Water and food were provided ad libitum. After a 1‐week acclimatization period, animals were randomly divided into four groups (*n* = 5 per group). No statistical method was used to predetermine sample size. The sample size was determined based on previous studies [21] and following the principles of the 3Rs (replacement, reduction, and refinement) [22]. All animals were randomly allocated to experimental groups. Rats were housed in metal cages with environmental enrichment to minimize stress, and all procedures were conducted between 08:00 and 11:00 a.m. Actions were taken to minimize animal suffering and to reduce the number of animals used. The animals were distributed into groups as follows: The control group (control) received a standard diet supplemented with 35% sucrose, without pharmacological treatment. The acetylsalicylic acid (ASA) group was fed the same diet and received ASA as treatment. The melatonin (MEL) group also received the standard diet with 35% sucrose and was treated with MEL. Finally, the experimental group was fed the standard diet supplemented with 35% sucrose and treated with calafate (*B. microphylla*) (BM).

All experimental procedures were conducted in accordance with the National Institutes of Health Guide for the Care and Use of Laboratory Animals (NIH Publications No. 8023, revised 1978) and approved by the Scientific Ethics Committee of the University of La Frontera (File No. 113_17).

### 2.2. Diets and Supplementation

Animals were fed a standard diet (Prolab® RMH 3000, Purina LabDiet® Inc., Saint Louis, Missouri, USA) supplemented with a 35% sucrose solution in the drinking water (Table 1). From the beginning of the intervention, the experimental group received a daily dose of 350 mg/kg freeze‐dried calafate (BM) dissolved in drinking water, with an additional 20% added to compensate for potential degradation and residue (Table 2). The product was obtained from the company Patagonia Super Fruits, located in Coyhaique, Chile. The mixture was prepared daily to avoid oxidation of the bioactive compounds, and the bottles were wrapped in aluminum foil to prevent any possible effects of the product when exposed to light. The human‐equivalent dose, calculated based on body surface area and following the FDA‐recommended conversion factor, corresponds to 56.7 mg of freeze‐dried calafate for a 60‐kg adult human body weight [23]. The average daily fluid intake was estimated at approximately 80 mL per animal.

**Table 1:** Composition of the diet used in experiments.

Composition	Weight	Energy density	Energy
	%	kcal/kg	%
Proteins (methionine, L lysine)	22.5	1040	26
Lipids (soybean oil)	12.9	1350	15
Carbohydrates (starch, sucrose)	51.2	2360	59
Fibers	4.1	—	—
Vitamins/minerals	4.6	—	—

*Note:* Energy density: 3 kcal/g. Produced by Purina LabDiet®, Inc. [Prolab® RMH 3000].

**Table 2:** Nutritional composition of calafate (*Berberis microphylla*).

Nutritional contribution	100 g
Energy (kcal)	246
Protein (g)	10.1
Carbohydrates (g)	48.5
Fat (g)	1.3
Fiber (g)	30.5

*Note:* Nutritional information is shown per 100 g of freeze‐dried product. Produced by the company Patagonia Super Fruits®, Coyhaique, Chile.

**Figure 1:**
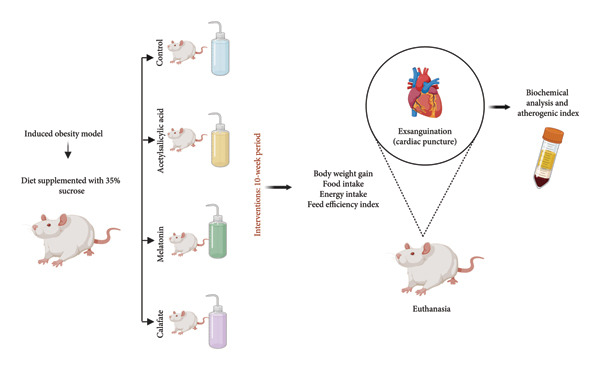
General diagram of the research. Rats were fed a diet supplemented with 35% sucrose. This established the obesity induction model. The animals were divided into 4 groups: one control, two positive controls, and one experimental group supplemented with calafate. The protocol lasted 10 weeks.

The antioxidant capacity, as well as the total polyphenol and anthocyanin contents of the freeze‐dried calafate, had been previously characterized by the same research group [24]. Considering the proinflammatory state associated with obesity, two positive controls were included: MEL and ASA. MEL, known for its potent antioxidant properties, was administered at a dose of 10 mg/kg/day, dissolved in the drinking water [25]. Similarly, ASA, selected for its anti‐inflammatory efficacy, was administered at the same dose and route [26]. To preserve the stability of the bioactive compounds, all treatment solutions were freshly prepared each day. The intervention lasted 10 weeks [27]. The summary of the research protocol is presented in Figure 1.

### 2.3. Experimentation and Euthanasia

During the experimental protocol, body weight was measured weekly at a consistent time using a precalibrated digital balance (Measuretek EHW‐EC, resolution of 0.5 g, Shanghai, China). Solid food intake was recorded daily using an analytical balance, and liquid intake was measured with a 200‐mL graduated cylinder. Both parameters were reported as mean values per cage, with two animals housed per cage. Energy intake was estimated based on individual solid food consumption. Feed efficiency index, body weight gain, and cumulative food intake were calculated according to standard procedures [28].

At the end of the 10‐week intervention, animals were fasted for 8 h prior to euthanasia. Euthanasia was performed using gaseous isoflurane saturation in a dedicated chamber, with unconsciousness confirmed by an experienced veterinarian through visual inspection. Subsequently, exsanguination was conducted via cardiac puncture with the animal positioned in dorsal recumbency. Blood samples were collected into lithium heparin tubes and centrifuged at 3000 rpm for 15 min at 4°C. The plasma samples were aliquoted and stored at −80°C for further analyses.

### 2.4. Biochemical Analysis and Atherogenic Index

As this study builds on our previously published work, biochemical analyses were performed following the same laboratorial methodology described in that research [16]. In summary, plasma concentration of glucose, total cholesterol, HDL‐c, and TG were measured using commercial enzymatic kits (Biosystems®, Recife, Pernambuco, Brazil). Quantifications were performed spectrophotometrically using a Snibe Biossays 240 Plus® microplate reader (Mumbai, Maharashtra, India). Very‐low‐density lipoprotein cholesterol (VLDL‐c) was estimated using the equation VLDL‐c = TG/5 [29]. Atherogenic indices were calculated as follows: Castelli Risk Index I (total cholesterol/HDL‐c), plasma atherogenic index (log_10_ [TG/HDL‐c]), atherogenic coefficient ([total cholesterol − HDL‐c]/HDL‐c), and the TyG index (ln [TG × glucose]/2) [29].

### 2.5. Feed Conversion Efficiency

Feed conversion efficiency was calculated as body weight gain (g) divided by total feed intake (g) during the experimental period. This method is widely used in dietary intervention studies in animal models to assess the efficiency with which feed is converted into body mass [30].

### 2.6. Statistical Analysis

Statistical analyses were performed using the IBM SPSS Statistics, Version 21 (Endicott, New York, USA). Data normality was assessed with the Shapiro–Wilk test. Comparisons between groups were performed using one‐way analysis of variance (ANOVA), followed by Tukey’s post hoc test for multiple comparisons. Results are expressed as mean ± standard deviation (SD). Differences were considered statistically significant at *p* < 0.05.

## 3. Results

### 3.1. Dietary Composition and Intake Behavior

The composition of the standard diet used in this study is presented in Table 1. It provided an energy density of 3 kcal/g, with carbohydrates accounting for 59% of the total caloric value. As shown in Figures 2(a), 2(b), all experimental groups (ASA, MEL, and BM) demonstrated a significant increase in solid food intake compared to the control group (*p* < 0.05), with the highest intake observed in the ASA group. Similarly, liquid intake was significantly higher in the BM group relative to all other groups (*p* < 0.05, Figures 2(c), 2(d)).

Figure 2Dietary variables and body weight. (a) Solid intake in experimental groups during the 10‐week intervention; (b) solid intake mean for experimental groups; (c) liquid intake in experimental groups during the 10‐week intervention; (d) liquid intake mean for experimental groups; (e) calorie intake for experimental groups. Calorie intake was calculated based on the energy density of the solid intake and liquid intake; (f) food efficiency ratio for the 10‐week intervention; (g) body weight in experimental groups during the 10‐week intervention; (h) average body weight increase. ASA: acetylsalicylic acid. MEL: melatonin. BM: freeze‐dried calafate (*Berberis microphylla*). *n* = 5/group. Data are represented as mean ± standard deviation. Mean values with different letters indicate a significant difference (*p* < 0.05).

**Figure:**
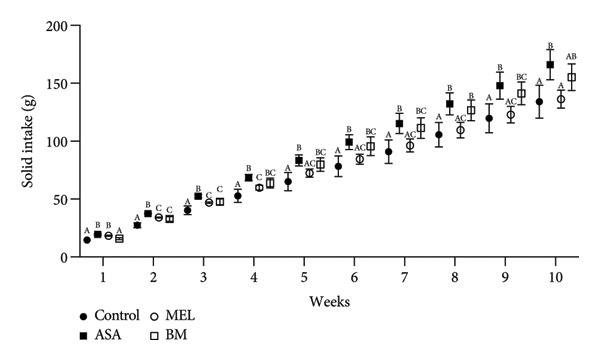
(a)

**Figure:**
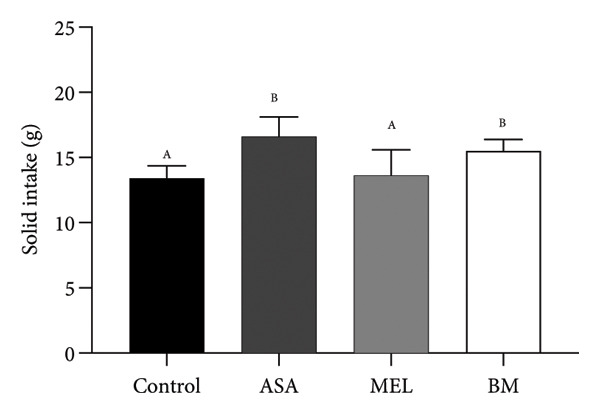
(b)

**Figure:**
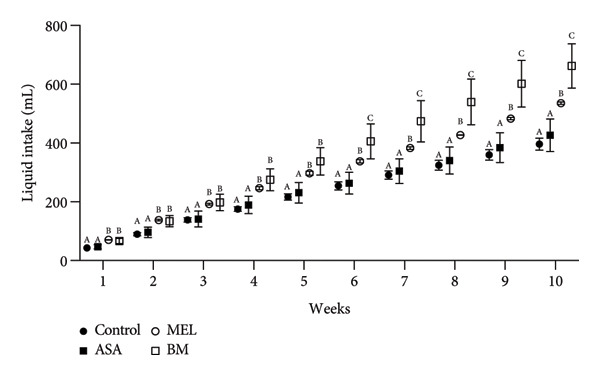
(c)

**Figure:**
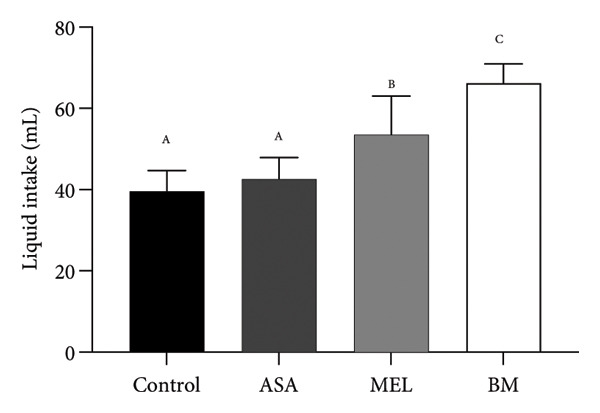
(d)

**Figure:**
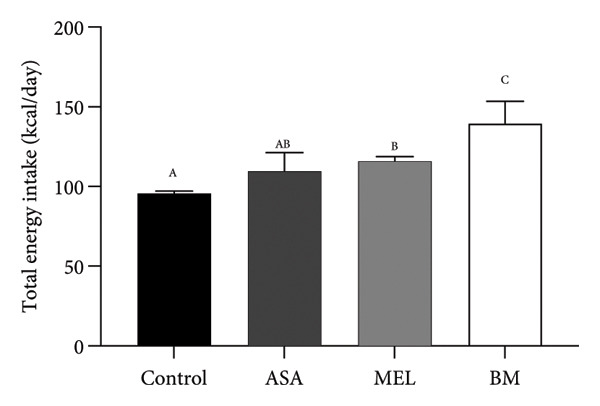
(e)

**Figure:**
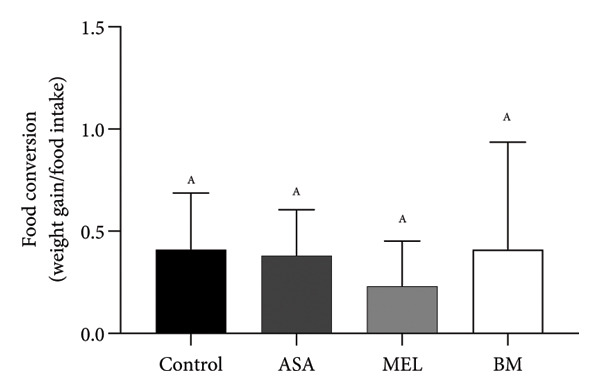
(f)

**Figure:**
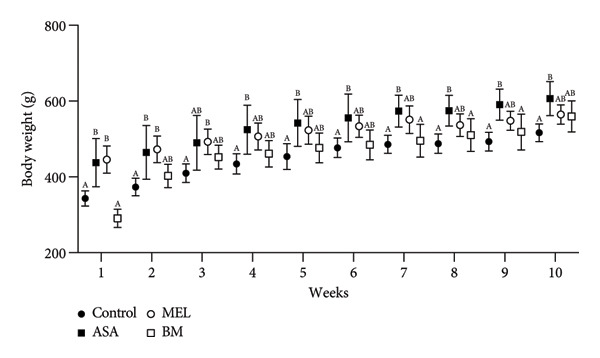
(g)

**Figure:**
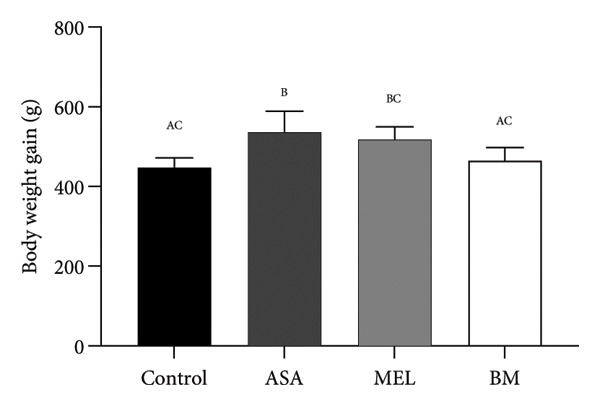
(h)

This pattern of increased consumption was reflected in total energy intake (Figure 2(e)), which was significantly elevated in the BM group compared to the control and MEL (*p* < 0.05) groups. However, no significant differences were observed in feed conversion efficiency among groups (Figure 2(f)). The energy contribution of freeze‐dried calafate was not considered in the calculation of total intake, since the amount administered corresponded to less than 2% of the animal’s daily caloric intake.

### 3.2. Body Weight and Weight Gain

Despite increased caloric intake, the BM group exhibited a trend toward lower body weight gain compared to the ASA and MEL groups (Figures 2(g), 2(h)). Notably, the ASA group showed the highest weight gain, while both the control and BM groups demonstrated significantly lower final weight gain (*p* < 0.05).

### 3.3. Plasma Biochemical Parameters

As shown in Figure 3(a), all treated groups exhibited significantly lower plasma glucose concentrations compared to the control group (*p* < 0.05), with the BM group showing a reduction comparable to the MEL group. Total cholesterol levels were significantly elevated in all treated groups (ASA, MEL, and BM) relative to the control group (Figure 3(b)). However, only the MEL group exhibited significantly higher plasma TG (Figure 3(c)), while the BM group maintained levels similar to the control group. VLDL‐c levels (Figure 3(d)) were increased in the MEL group compared to the control group, with no significant difference observed in the BM group. HDL‐c was significantly increased in all treated groups (Figure 3(e)), particularly in the ASA group.

Figure 3Plasma parameters. (a) Glucose (mg/dL) mean for experimental groups; (b) total cholesterol (mg/dL) mean for experimental groups; (c) triglycerides (mg/dL) mean for experimental groups; (d) VLDL cholesterol (mg/dL) mean for experimental groups; (e) HDL cholesterol (mg/dL) mean for experimental groups. ASA: acetylsalicylic acid. MEL: melatonin. BM: freeze‐dried calafate (*Berberis microphylla*). *n* = 5/group. Data are represented as mean ± standard deviation. Mean values with different letters indicate a significant difference (*p* < 0.05).

**Figure:**
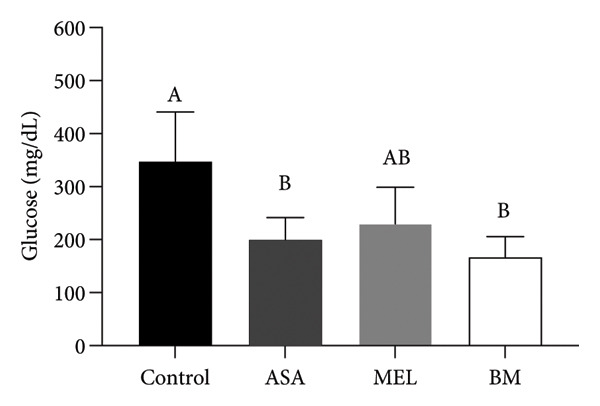
(a)

**Figure:**
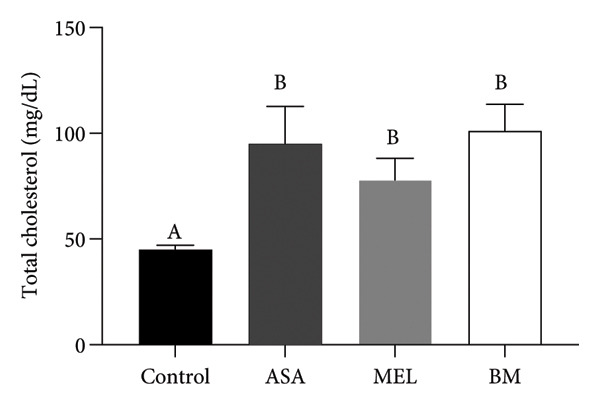
(b)

**Figure:**
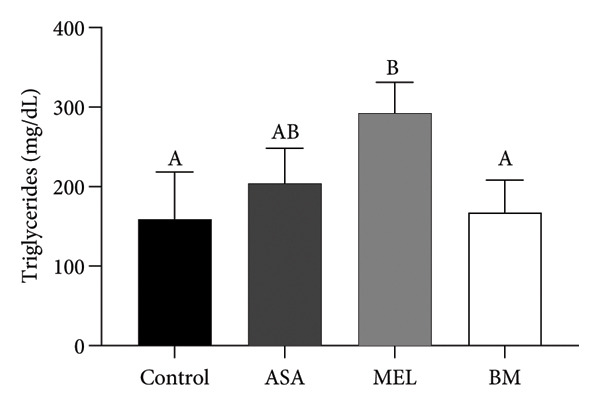
(c)

**Figure:**
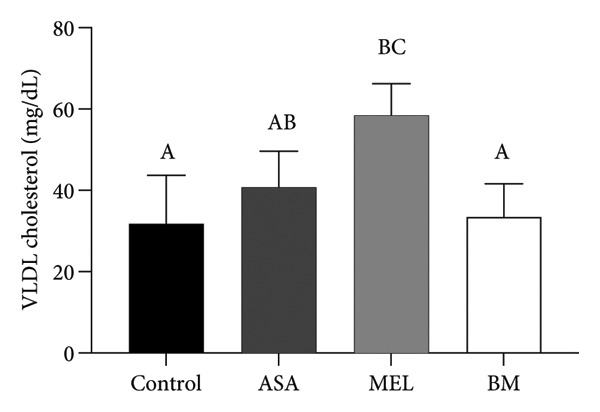
(d)

**Figure:**
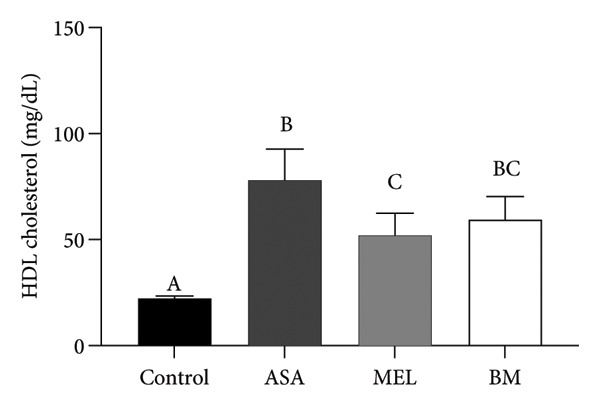
(e)

### 3.4. Atherogenic Indices

Figure 4 and Table 3 summarize the effects of treatments on plasma atherogenic markers. The BM group exhibited a significant reduction in the atherogenic index (log_10_[TG/HDL‐c]) compared to the control (*p* < 0.05), as well as in the TyG index. Although the Castelli index and atherogenic coefficient remained elevated compared to ASA, the BM group showed intermediary values between control and MEL groups, indicating partial modulation. According to Table 3, the BM group reduced the atherogenic index by 49.1% and the TyG index by 2.94% relative to the control.

Figure 4Atherogenic index. (a) Castelli index mean for experimental groups; (b) atherogenic coefficient mean for experimental groups; (c) atherogenic index mean for experimental groups; (d) glucose–triglyceride index mean for experimental groups. ASA: acetylsalicylic acid. MEL: melatonin. BM: freeze‐dried calafate (*Berberis microphylla*). *n* = 5/group. Data are represented as mean ± standard deviation. Mean values with different letters indicate a significant difference (*p* < 0.05).

**Figure:**
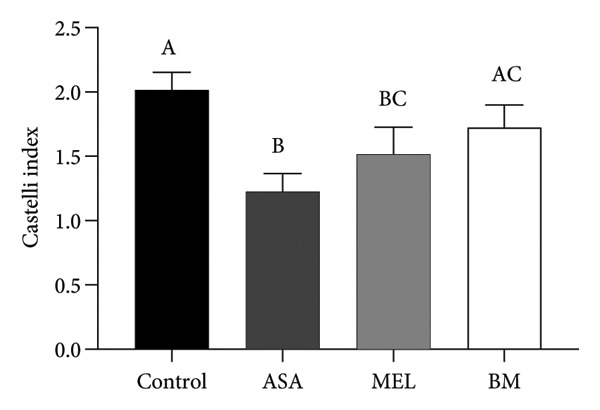
(a)

**Figure:**
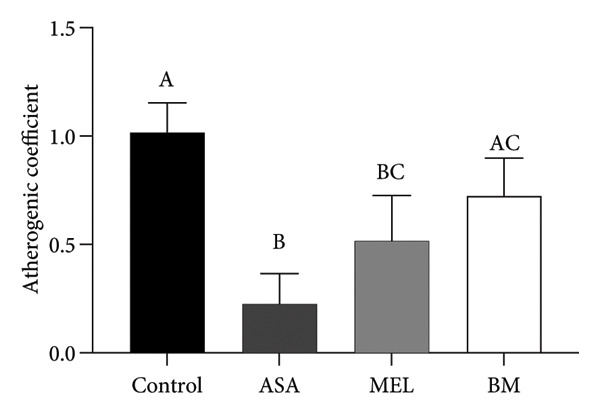
(b)

**Figure:**
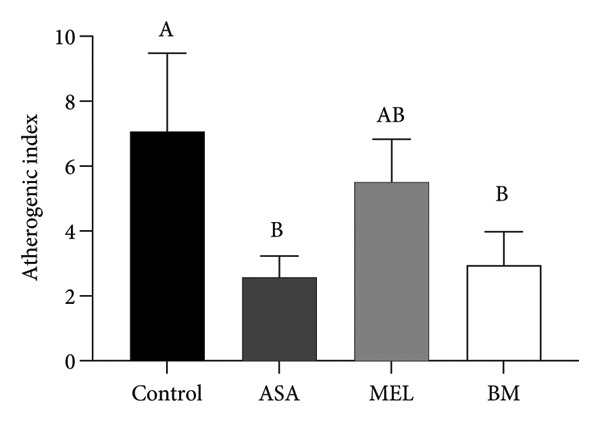
(c)

**Figure:**
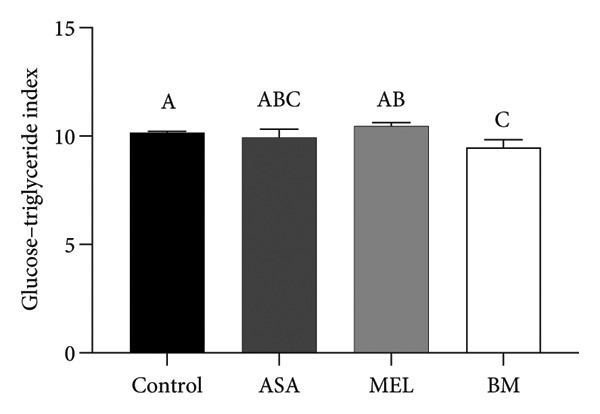
(d)

**Table 3:** Percentage change of plasma atherogenic indexes.

Composition	Weight	Energy density	Energy
	%	kcal/kg	%
Castelli index^#^	↓14.5	↓39.2	↓24.7
Atherogenic index^∃^	↓28.8	↓77.8	↓49.1
Atherogenic coefficient^∀^	↓58.1	↓63.5	↓22.1
Triglyceride–glucose ratio^&^	↓6.80	↓2.16	↓2.94

*Note:* Data are represented as percentage change (%) (↓: decrease or ↑: increase) of plasma atherogenic indexes of groups supplemented with freeze‐dried calafate (*Berberis microphylla*) (BM), acetylsalicylic acid (ASA), and melatonin (MEL), with respect to the control group.

^#^(Total cholesterol/HDL cholesterol).

^∃^(log10 [TG/HDL cholesterol]).

^∀^([Total cholesterol − HDL cholesterol]/HDL cholesterol).

^&^(ln [triglycerides × glucose]/2).

## 4. Discussion

This study demonstrated that supplementation with freeze‐dried calafate (BM) in rats subjected to a high‐sucrose diet was associated with favorable modifications in plasma lipid profile and atherogenic indices, despite a significant increase in total caloric intake. This may be due to a modulating effect of calafate on adiposity and metabolic efficiency, which must be confirmed in future studies, with measurement of adipose tissue. Moreover, the BM group showed an increase in HDL‐c levels and a reduction in TG and VLDL‐c, resulting in improved composite CVR markers, including atherogenic index and TyG ratio. These findings highlight the potential of calafate as a functional food capable of attenuating diet‐induced metabolic disturbances and reducing early CVR. Dyslipidemia and diets based on ultraprocessed foods, particularly those high in simple carbohydrates and low in fiber, are recognized as major contributors to the development of CVDs [31]. Although the Food and Drug Administration (FDA) recommends limiting simple sugar intake to 50 g/day, global consumption averages is 73 g/day, with some Latin American countries such as Chile reporting values as high as 158 g/day [32]. To mimic these dietary patterns, animal models using high‐carbohydrate diets have been widely adopted, as they reproduce the metabolic profile observed in populations with excessive sugar intake [33, 34]. Evidence shows that sugar‐enriched diets, especially those containing sugar‐sweetened beverages, are associated with hyperglycemia, increased fatty acid oxidation, and upregulation of cholesterol biosynthesis pathways [35]. Such alterations contribute to the onset of hypertriglyceridemia and elevated blood glucose, reinforcing their role in the pathogenesis of CVDs [36]. In this context, the search for dietary compounds capable of modulating lipid metabolism and reducing atherogenic risk has intensified [37]. Among these, berries have attracted increasing attention due to their high content of antioxidant phytochemicals, particularly polyphenols and anthocyanins, which have been linked to cardioprotective effects [38]. The use of composite atherogenic indices has emerged as a more sensitive approach to assess CVR compared to traditional lipid measurements [39, 40]. Indices such as the Castelli Risk Index I (total cholesterol/HDL‐c), the atherogenic coefficient ([total cholesterol − HDL‐c]/HDL‐c), and the atherogenic index of plasma (log_10_[TG/HDL‐c]) are considered strong predictors of atherogenesis and cardiovascular events. These indices integrate multiple lipid parameters, enhancing their predictive value for subclinical inflammation, insulin resistance, and endothelial dysfunction. Several studies have demonstrated that these ratios are more closely associated with cardiovascular outcomes than isolated markers like LDL‐c or TG [41]. In animal models, interventions that modulate these indices are often linked to improved vascular reactivity, reduced lipid peroxidation, and attenuation of inflammatory pathways. Despite their clinical relevance, few studies have explored the effect of functional foods, especially polyphenol‐rich fruits like calafate, on these specific indices under conditions of diet‐induced obesity [42–44]. The present study evaluated the effect of calafate (*B. microphylla*) supplementation on the lipid profile and atherogenic indices in rats with obesity induced by a high‐sucrose diet. The freeze‐dried fruit used in this protocol exhibited a higher total phenolic content than that reported for other native berries such as murtilla (*Ugni molinae* Turcz) and bilberry (Vaccinium corymbosum) [24]. In light of previous findings, calafate also demonstrated similar antioxidant capacity and a rich profile of phenolic compounds, comparable to fruits like maqui (*Aristotelia chilensis*), which are known for their ability to inhibit lipid peroxidation [45]. These biological properties have been linked to the presence of anthocyanins, flavonoids, hydroxycinnamic acids, and proanthocyanidins. After 10 weeks of continuous administration, animals in the BM group showed a significantly higher liquid intake compared to the control and both the positive control groups (MEL and ASA), contributing to an overall increase in energy intake. However, no significant difference in body weight was observed between the BM and control groups. This finding suggests that the modulation of weight gain in the BM group is not attributable to reduced food intake, as food efficiency remained similar between the two groups. Therefore, mechanisms other than appetite suppression, possibly involving energy metabolism, lipid oxidation, or inflammatory modulation, may underlie the absence of excessive weight gain despite increased caloric consumption. On the other hand, it is important to note that the astringency characteristic of fruits rich in polyphenols, such as calafate (*B. microphylla*), could influence palatability and, consequently, voluntary fluid intake. Previous studies have shown that the astringency attributed to polyphenols, particularly tannins and anthocyanins, can modify sensory perception and affect intake in both animal models and humans [46]. In our model, however, the animals in the BM group did not reduce their consumption but rather increased it significantly compared to the controls. This suggests that the astringency of calafate did not act as a limiting factor; on the contrary, it may have induced changes in palatability or interaction with the sucrose solution used as a vehicle, increasing total fluid intake. This allows us to consider astringency as a sensory factor that should be taken into account in the design of future interventions with fruits rich in polyphenols. Supporting this hypothesis, Olivares‐Caro et al. demonstrated that calafate extract was capable of downregulating TNF‐α gene expression in murine macrophages and reducing lipid peroxidation in vivo. Additionally, chronic supplementation in high‐fat diet–fed animals led to increased adiponectin levels and favorable changes in thrombomodulin expression, indicating vascular protection and anti‐inflammatory effects [18]. Although the present study did not directly evaluate inflammatory or molecular markers, the observed improvements in the atherogenic indices and lipid profile may, at least in part, reflect the action of these bioactive pathways. The exact mechanism by which this occurs is unknown; however, previous studies have described approaches that indicated that supplementation with polyphenol‐rich food sources in animal models generates an increase in brown adipose tissue, elevating its thermogenic properties and, therefore, causing an increase in energy expenditure. Duarte et al. demonstrated that acute administration of calafate increased the relative expression of the protein iodothyronine deiodinase Type 2​ (Dio2), a marker of thermogenesis, and prevented alterations in mitochondrial crests and extracellular matrix composition induced by consumption of a high‐fat diet [47]. Supplementation with nutritional polyphenols was sufficient to decrease rats’ body weight gain by 7%, having enough relevance to cause substantial metabolic improvements [48]. Differences found between the association of the high‐sucrose diet supplemented with calafate and the controls indicate the possibility that the active components of the berry modulate weight; however, this cannot be confirmed. It is of interest to further study the physiological implications of the relationship between calafate (*B. microphylla*) and/or its components in obesity. Regarding plasma parameters, it has been described that different sources that are high in antioxidant components, such as pomegranate (*Punica granata*) and cinnamon (*Cinnamomum verum*), decrease glucose concentrations in animals supplemented with such products [49, 50], which is also observed in this research, where calafate consumption showed a significant decrease in plasma glucose by 52% compared to controls, and 16.6% and 27.5% compared to ASA and MEL, respectively, although without showing statistically significant differences with respect to positive controls, suggesting that calafate attenuated physiological parameters through the regulation of blood glucose homeostasis in obese rats. Further studies are warranted to elucidate the precise molecular mechanisms by which calafate exerts its cardiometabolic effects. This regulation is produced by a mechanism that has not yet been fully explained, although it may be associated with the facilitation of glucose catabolism due to induction of the glycolysis pathway in the cell or by inhibition of the enzyme alpha‐glucosidase [51, 52]. It is known that polyphenols present in food can reach concentrations in the intestine and can interact with α‐amylase and α‐glucosidase enzymes, modifying glycemic responses by inhibiting carbohydrate digestion [20]. The greatest CVR is conferred by the simultaneous presence of low HDL‐c and high TG; in this context, hypercholesterolemia is closely related to the pathogenesis of atherosclerosis [31]. The effect on the lipid profile in rats supplemented with calafate showed a significant increase in total cholesterol compared to the control, which is explained by the increase in HDL‐c concentrations, which increased in the BM versus control and BM versus MEL groups by 166% and 14%, respectively. In previous research, in an effort to obtain a marker of CVR, the calculation of atherogenic index has been proposed, since they have shown correlation with the size of lipoprotein particles and the increase in LDL [51, 52]. Furthermore, extracts from the calafate fruit have been shown to exert modulatory effects on fat accumulation, thermogenesis, and metabolic status in experimental models. For example, Duarte et al. showed that supplementation with a calafate extract (50 mg of polyphenols/kg weight) in C57BL/6J mice on a high‐fat diet significantly reduced body weight gain, with an increase in energy expenditure and promotion of white adipose tissue browning and expression of thermogenic markers such as uncoupling protein‐1 (UCP‐1), peroxisome proliferator‐activated receptor alpha (Pparα), peroxisome proliferator‐activated receptor gamma coactivator‐1 alpha (Pgc1α), the Prdm16 gene transcription factor, NAD‐dependent sirtuin‐1 deacetylase (Sirt1), and the enzyme iodothyronine 5′‐deiodinase, iodothyronine 5′‐monodeiodinase (Dio2) [46]. In another study, calafate extract also improved mitochondrial function in brown adipose tissue, reversing the decrease in UCP‐1 induced by a high‐fat diet, increasing the expression of mitochondrial fusion proteins such as Opa1, and increasing basal energy activity [53]. Additionally, in cell models, calafate has shown anti‐inflammatory effects in adipocyte–macrophage interaction, specifically, calafate extracts inhibit lipopolysaccharide (LPS)‐induced nitric oxide secretion, decrease the expression of inducible nitric oxide synthase (iNOS) and tumor necrosis factor alpha (TNF‐α), and induce interleukin 10 (IL_10_) in cocultures of 3T3 L1 adipocytes and RAW264.7 macrophages, suggesting a mitigation of the inflammatory state linked to adiposity [54]. Overall, the evidence indicates that calafate has the potential to promote weight loss or containment of adiposity, acting through at least three interrelated mechanisms: increased energy expenditure, induction of thermogenesis/browning, and reduction of inflammation in adipose tissue [55]. However, the magnitude of the effects appears to depend on the duration of treatment, the dose of the extract/polyphenols, and the basal metabolic state of the model (e.g., degree of obesity and diet). In our study, calafate supplementation was linked to a significant reduction in the atherogenic index and the TyG index compared to the control groups. This suggests a potential antiatherogenic effect of calafate (*B. microphylla*), indicating a lowered CVR. However, there are limitations to our study. Therefore, it is essential to conduct further research with a larger sample size and additional experimental analyses to better understand the mechanisms behind these results, such as hepatic TG and fecal caloric content. Nevertheless, the study presents limitations, including a relatively small sample size and the absence of complementary analyses that could elucidate the underlying mechanisms, such as oxidized LDL quantification and inflammatory biomarkers. The effects cannot be attributed solely to polyphenols because freeze‐dried fruit was used, not just polyphenols. Future research should aim to expand the sample size and incorporate additional metabolic assessments, including hepatic TG content and fecal energy loss, to better characterize the physiological pathways involved in the observed effects.

## 5. Conclusions

The present study demonstrates that calafate (*B. microphylla*) supplementation may reduce CVR by improving plasma lipid homeostasis, particularly through increased HDL‐c levels and reductions in atherogenic index and the TyG ratio. These findings support the hypothesis of a potential antiatherogenic effect, reinforcing the role of calafate as a functional food with cardioprotective properties.

## Ethics Statement

The research was conducted in accordance with the National Institutes of Health Guide for the Care and Use of Laboratory Animals (NIH Publications No. 8023, revised 1978) and was approved by the Scientific Ethics Committee of the University of La Frontera (File No. 113_17).

## Consent

The authors have nothing to report.

## Disclosure

All authors have read and agreed to the published version of the manuscript.

## Conflicts of Interest

The authors declare no conflicts of interest.

## Author Contributions

Carla Guzmán‐Pincheira: conceptualization, methodology, formal analysis, investigation, resources, writing–original draft preparation, writing–review and editing, supervision, project administration, and funding acquisition. Gabriel Araujo‐Silva: validation, formal analysis, investigation, writing–original draft preparation, and writing–review and editing. Raul Sánchez‐Gutiérrez: conceptualization, methodology, formal analysis, investigation, writing–original draft preparation, writing–review and editing, and supervision.

## Funding

The authors report no funding.

## Data Availability

The data that support the findings of this study are available from the corresponding author upon reasonable request.
